# Disrupting the Interaction between Retinoblastoma Protein and Raf-1 Leads to Defects in Progenitor Cell Proliferation and Survival during Early Inner Ear Development

**DOI:** 10.1371/journal.pone.0083726

**Published:** 2013-12-31

**Authors:** Wenyan Li, Shan Sun, Yan Chen, Huiqian Yu, Zheng-Yi Chen, Huawei Li

**Affiliations:** 1 Department of Otorhinolaryngology, Affiliated Eye and ENT Hospital of Fudan University, Shanghai, China; 2 Research Center, Affiliated Eye and ENT Hospital of Fudan University, Shanghai, China; 3 Institute of Stem Cell and Regeneration Medicine, Institutions of Biomedical Science, Fudan University, Shanghai, China; 4 State Key Laboratory of Medical Neurobiology, Fudan University, Shanghai, China; 5 Department of Otolaryngology and Program in Neuroscience, Harvard Medical School and Eaton Peabody Laboratory, Massachusetts Eye and Ear Infirmary, Boston, Massachusetts, United States of America; Cardiological Center Monzino, Italy

## Abstract

The retinoblastoma protein (pRb) is required for cell-cycle exit of embryonic mammalian hair cells but is not required for hair cell fate determination and early differentiation, and this provides a strategy for hair cell regeneration by manipulating the pRb pathway. To reveal the mechanism of pRb functional modification in the inner ear, we compared the effects of attenuated pRb phosphorylation by an inhibitor of the Mitogen-Activated Protein (MAP) kinase pathway and an inhibitor of the Rb–Raf-1 interaction on cultured chicken otocysts. We demonstrated that the activity of pRb is correlated with its phosphorylation state, which is regulated by a newly established cell cycle-independent pathway mediated by the physical interaction between Raf-1 and pRb. The phosphorylation of pRb plays an important role during the early stage of inner ear development, and attenuated phosphorylation in progenitor cells leads to cell cycle arrest and increased apoptosis along with a global down-regulation of the genes involved in cell cycle progression. Our study provides novel routes to modulate pRb function for hair cell regeneration.

## Introduction

In vertebrates the inner ear mediates multiple sensory inputs, including sound, balance, and acceleration. This complex sensory organ begins its development as a bilateral thickening of the surface ectoderm, regarded as the otic placode, which develops lateral to the developing hindbrain. The developing placode descend beneath the surface ectoderm to form the otocyst [1]. Since carrying the genetic information required for the development of most cell types and structures of inner ear [2]–[4], chicken otocysts can be explanted from the developing embryo and this provides special opportunities for the in vitro analysis of the molecular mechanisms behind cellular proliferation and differentiation in the inner ear.

It has been shown that retinoblastoma protein (pRb), encoded by the retinoblastoma gene *Rb1*, is required for proper cell cycle exit in the developing mouse inner ear, and its deletion in the embryo leads to proliferation of the sensory progenitor cells that differentiate into hair cells and supporting cells [5]. However, the role of pRb in proliferative progenitor cells during early development of the inner ear has not been established. In addition to its essential role in cell cycle exit, pRb also plays a crucial role in hair cell survival [5]–[7]. Therefore, modulation of pRb function instead of permanent *Rb1* gene deletion is an attractive route through which cell proliferation and survival might be achieved for hair cell regeneration [8].

The function of pRb is correlated with its phosphorylation state, and a cell cycle-dependent pathway mediated by the Mitogen-Activated Protein (MAP) kinase cascade plays a role in maintaining the phosphorylation state of pRb. The activation of this cascade leads to up-regulation of cyclin E/cdk2 or cyclin D/cdk4 kinase activity that, in turn, induces pRb phosphorylation. Sufficient pRb phosphorylation inactivates its transcriptional repressor function, and this allows for the expression of E2F target genes [9]. The mechanisms of pRb inactivation and subsequent effects are species, tissue, and cell-type specific, but the general role of MAP kinase on pRb phosphorylation during the early development of the inner ear is still unclear. In addition to the MAP kinase cascade, it has recently been shown that Raf-1 physically interacts with pRb to regulate its function early in the G1 phase and this interaction serves as a link between mitogenic signaling and cell cycle regulation [10], [11]. Disruption of the pRb–Raf-1 interaction induces apoptosis in malignant tumor cells and inhibits cell proliferation [11]–[14]. Whether the pRb–Raf-1 interaction is involved in the regulation of pRb during early inner ear development has yet to be determined.

We used cultured chicken otocysts to investigate the proliferation, apoptosis, and differentiation of progenitor cells in response to pharmacological modulation of pRb function. Inhibitors that target different pathways that regulate pRb phosphorylation were used to reveal the molecular mechanisms behind this regulation. This study provides new opportunities for hair cell regeneration by modulating pRb function.

## Materials and Methods

### Chicken Embryos

Fertilized eggs from a breeding chicken farm (Guixing, Shanghai) were incubated in a humidified incubator maintained at 38°C until they reached the desired stages according to the criteria of Hamburger and Hamilton [15]. The Animal Care and Use Committee of Fudan University approved all animal procedures.

### Otocyst Culture and Treatment

Embryos at stage HH16–18 were exposed by breaking the air cell of eggs, then immersed in 0.02% Tricaine (Sigma, St. Louis, MO) until the whole embryo is still and without any movement. The otocysts were dissected in phosphate-buffered saline (PBS, pH 7.2) from the surrounding mesenchymal tissue with delicate ophthalmic forceps under a dissection microscope. The dissected otocysts were treated with trypsin (0.125% in PBS) at room temperature for 30 s to remove any residual periotic mesenchyme and rapidly transferred into 5 mL serum-free culture medium in a petri dish for floating culture at 37°C in a humidified atmosphere containing 5% CO_2_. The culture medium was composed of equal parts high-glucose Dulbecco’s modified Eagle’s medium (DMEM) and F12 medium supplemented with N2 and B27 (media and supplements were from Invitrogen/GIBCO/BRL, Carlsbad, CA) and 50 IU/mL penicillin [16], [17].

The explanted otocysts were treated with various concentrations of the Rb–Raf-1 inhibitor RRD-251 (Sigma, St. Louis, MO), and the MEK inhibitor U0126 (Sigma) for 24 h. Otocysts cultured in medium with 0.1% DMSO were used as vehicle-only controls, and the DMSO had no observable effects on cell survival or proliferation. For immunostaining, cultured otocysts were harvested and fixed for 0.5 h in 4% (w/v) paraformaldehyde (PFA) at 4°C. For western blot analysis and quantitative RT-PCR, the otocysts were flash-frozen in liquid nitrogen and kept at −80°C for the following experiments.

### Quantitative RT-PCR

RNA was obtained from pooled otic vesicles either from chicken embryos at developmental stages HH18 (n = 40), HH20 (n = 25), HH24 (n = 20), and HH27 (n = 10) or from cultured otocyst pools from control and U0126- and RRD-251-treated groups with the AllPrep DNA/RNA/Protein Mini kit (Qiagen, Valencia, CA) following the manufacturer’s protocol. Purified mRNA was reverse-transcribed with the PrimeScript RT-RCR kit (TaKaRa Co., Dalian, China), and real time PCR was performed using the Sybr Green Premix Ex Taq kit (TaKaRa Co.). Each quantitative real-time PCR (qRT-PCR) run used cDNA generated from 20 ng of RNA. Primers were designed to have comparable melting temperatures of around 60°C and, when possible, to span exon-exon junctions. β-actin was used for calibration. The PCR reaction for each gene was set up in triplicate. The data presented here are the averages of at least two independent experiments, and the fold change in expression levels was determined using the DMSO-treated samples as the control. The estimated level of gene expression was calculated as 2^−ΔΔCt^ and statistical significance was analyzed using one-way ANOVA. Primer sequences were listed in Table 1.

**Table 1:** Primers used in the study.

GeneName	Forward Primer	Reverse Primer
*Ccnb2*	5′-gcatcaaaccaccagtaaagg-3′	5′-ggagcaacacatcagagaagg-3′)
*Ccnb3*	5′-atcaccaacgctcacaagaac-3′	5′-ctcaggctccacaggaacat-3′
*Ccnd3*	5′-atgccccttactgtggagaag-3′	5′-gatggagaatgtgagccaaga-3′
*Ccne1*	5′-tgggcaaacagagatgatgta-3′	5′-cacaaacctccattagccagt-3′
*Ccne2*	5′-ctgaagaaggagaaccgatacg-3′	5′-ggaggcaatgaagagtgaggta-3′
*Cdc2*	5′-tctgctctgtattccactcctg-3′	5′-attgttgggtgtccctaaagc-3′
*Myb*	5′-tacccctactaccacattgctg-3′	5′-gccctttcagttcattctcagt-3′
*Myc*	5′-ctgaagcgaacgagtctgaat-3′	5′-agcgtagttgtgttggtggat-3′
*Raf-1*	5′-gaaaataggagactttggtctagc-3′	5′-atctgactgaaaactgaacgga-3′
*Rb1*	5′-ggacagggatgtgctgagattg-3′	5′-tgccataggtagccatgacaat-3′
*β-actin*	5′-gatggactctggtgatggtgttac-3′	5′-ttgatgtcacgcacaatttctctc-3′

### Western Blotting

Protein lysates were obtained from pooled otic vesicles from HH18, HH20, HH24, and HH27 chicken embryos or from cultured otocysts from control and U0126- and RRD-251-treated groups with the AllPrep DNA/RNA/Protein Mini kit (Qiagen). The relative amounts of total protein and the differences in concentration among the samples were determined with a BCA protein assay kit (Thermo Fisher Scientific, Rockford, IL). Proteins were separated on SDS-polyacrylamide gels and transferred to nitrocellulose membranes. The membranes were incubated with a blocking solution (5% non-fat milk in TRIS-buffered saline with 0.1% Tween-20 (TBS-T)) for 1 h at room temperature and then blotted overnight with primary antibodies at 4°C. The antibodies were diluted in blocking solution to analyze the levels of Raf-1 kinases (1∶500, BD Transduction Laboratories, Franklin Lakes, NJ), pERK/ERK (1∶500, Bioworld Technology, Louis Park, MN), pRb (1∶1000, Cell Signaling Technology, Danvers, MA), cleaved Caspase-3 (1∶500, Cell Signaling Technology, Danvers, MA), and β-actin (1∶5000, Sigma, St. Louis, MO). All the antibodies involved in our study were produced against highly conserved proteins from chick to mammal and the majority have been confirmed by other chicken studies [2], [16]. Unbound primary antibodies were removed by four washes of 15 min each in TBS-T at room temperature. Bound primary antibodies were detected with horseradish peroxidase-conjugated antibody against rabbit or mouse IgG (Amersham Pharmacia Biotech) at a dilution of 1∶10000 in TBS-T. Antibody binding was visualized by chemiluminescence substrate (Thermo Fisher Scientific) and exposed to X-ray film. The films were scanned and analyzed with ImageJ software (Wayne Rasband, National Institutes of Health, USA).

### Cell Proliferation Assay

In order to label proliferating cells, 10 mM 5-ethynyl-2′-deoxyuridine (EdU) was added to the culture medium for 30 min prior to the fixation. The EdU signals were detected with the Click-iT EdU cell proliferation kit (Invitrogen, Grand Island, NY) followed by antibody labeling according to the immunofluorescent staining protocol described below.

### Immunofluorescent Staining

The fixed otocysts were rinsed three times for 5 min at room temperature in PBS and blocked for 30 min with 5% heat-inactivated goat serum and 0.1% Triton-100 in PBS (PBS-T). The samples were incubated with primary antibodies at 37°C for 1 h and then at 4°C overnight. The primary antibodies were monoclonal anti-neuron-specific β-III tubulin (Tuj1) (Covance, Princeton, NJ) and anti-cleaved Caspase-3 (Cell Signaling Technology) diluted 1∶1000 and 1∶200, respectively, in PBS-T [2], [16]. After three washes with PBS-T, TRITC-conjugated goat anti-rabbit or goat anti-mouse secondary antibodies (Invitrogen) were added at a dilution of 1∶400 in PBS at 37°C for 1 h. Counterstaining with 4′, 6-diamidino-2-phenylindole, dihydrochloride (DAPI) allowed visualization of cell nuclei. The coverslipped slides were analyzed by confocal microscopy (Leica TCS SP5, Wetzlar, Germany). For quantitative studies, ImageJ software was used to calculate the areas of otic vesicle (OV) region and acoustic-vestibular ganglia (AVG) regions, as well as the EdU-positive and cleaved Caspase-3–positive cells. Prism4 software (GraphPad Software Inc., La Jolla, CA) was used for statistic analysis. At least six otocysts were assayed per condition from at least three independent experiments, and all of the data are presented as the mean ± SEM. *P*-values <0.05 were considered significant.

## Results

### Rb1 and Raf-1 are Expressed during Early Inner Ear Development

**Figure 1:**
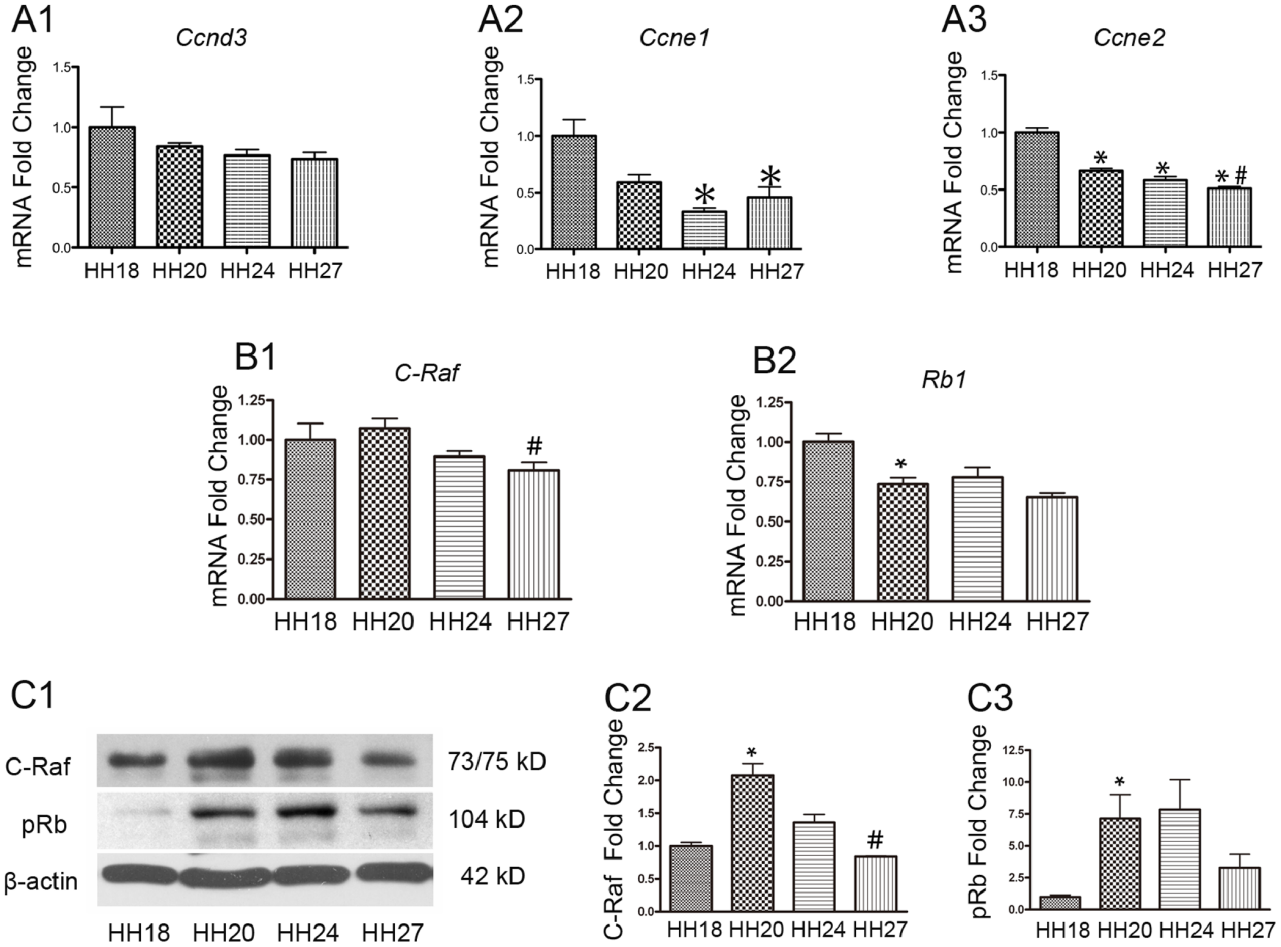
The expression of G1/S specific *cyclins*, *Rb1* and *Raf-1* during the development of otocyst. The expression of G1/S specific cyclins (A1-3), *Raf-1* and *Rb1* (B1-2) mRNA was analyzed by qRT-PCR at different embryonic development stages. Gene expression was normalized to the levels at stage HH18. (C1–C3) The protein lysates at different stages of inner ear development were analyzed by western blots to determine the levels of Raf-1 and pRb. β-actin was used as the loading control. At least three different experiments were evaluated, and statistical significance was estimated using one-way ANOVA. * = *P*<0.05 compared to HH 18, # = *P*<0.05 compared to HH 20.

Phosphorylation of pRb plays a role in the G1/S phase of cell cycle. We investigated G1/S specific cyclins during the development of inner ear, which served as good candidates to study their functional modification by pRb. We identified the expressions of G1/S specific genes *Ccnd3*, *Ccne1* and *Ccne2* were relatively higher during the stage HH18, while, *Ccne1* and *Ccne2* accordantly decreased during the further development (Fig. 1A).

The expression of chicken *Rb1* and *Raf-1* mRNA at specific stages was measured by qRT-PCR, and protein levels were measured by western blot. The expression of *Raf-1* mRNA was comparable at stages HH18, HH20, and HH24 but was downregulated at HH27 (Fig. 1B1). The presence of Raf-1 in the inner ear was confirmed by western blot at different development stages, and these protein levels had a similar expression pattern as *Raf-1* mRNA (Fig. 1C1 and C2).

The *Rb1* mRNA levels were highest at HH18 and then dropped off and were expressed at the same level in stages HH20–27 (Fig. 1B2). pRb protein was also detected at different stages. Based on the higher molecular weight and lower electrophoretic velocity, there was a significant amount of phosphorylated pRb present during early inner ear development (Fig. 1C1 and C3).

### The Phosphorylation of pRb is Required for the Proliferation of Developing Otocyst Cells through Two Independent Pathways

It has been reported that the Raf-1 kinase can bind and phosphorylate pRb early in the G1 phase, and this interaction has been investigated as a target for anticancer therapy [10], [13]. RRD-251 is a small molecule disruptor of the Rb–Raf-1 interaction that significantly inhibits angiogenesis and tumor growth both in vitro and in vivo in a pRb-dependent manner [12], [14]. We cultured otocysts in vitro in the presence of RRD-251 to further understand the role of Rb–Raf-1 interaction on pRb phosphorylation during early inner ear development. Further insight into the action of RRD-251 was obtained by studying its effects on cell proliferation in organotypic cultures of explanted otocysts. The morphological changes that occur in culture mimic the normal development of the inner ear, and the expression of the Tuj1 protein serves as a marker of neural processes. The cultured otocysts were divided into the OV (Tuj1-negative) and AVG (Tuj1-positive) areas that would develop into the sensory epithelium and the spiral ganglion, respectively.

**Figure 2:**
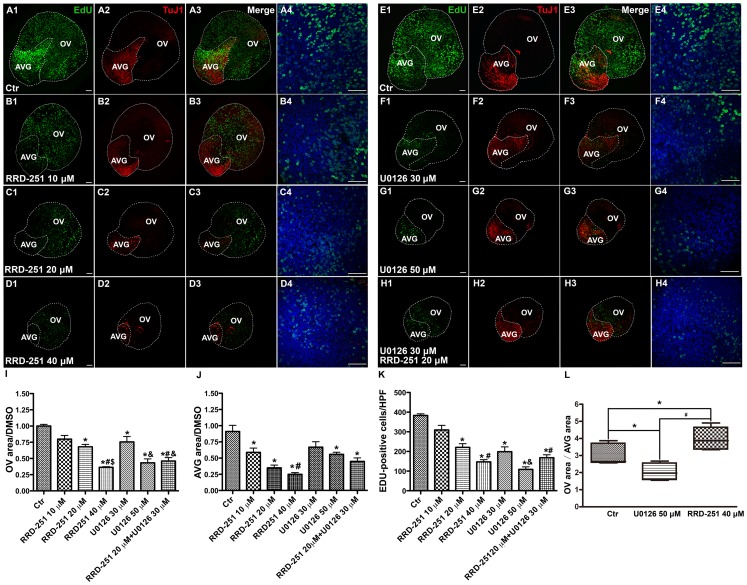
Cell proliferation was attenuated by selectively inhibiting the MAP kinase cascade and the Rb–Raf-1 interaction during the early development of the inner ear. Otocysts were explanted at HH16–18 and kept in culture medium with DMSO (A1–A4, E1–E4), RRD-251 10 µM (B1–B4), RRD-251 20 µM (C1–C4), RRD-251 40 µM (D1–D4), U0126 30 µM (F1–F4), U0126 50 µM (G1–G4), or a combination of RRD-251 20 µM and U0126 30 µM (H1–H4) for 24 h and then exposed to 10 mM EdU for 30 min. Cell proliferation is indicated by EdU (green) staining and the nuclei are counterstained with DAPI (blue). Based on the morphological changes that occur in culture that mimic the normal development of the inner ear and the expression of Tuj1 (red) that serves as a marker of neural processes, the cultured otocysts were divided into the otic vesicle area (OV, Tuj1-negative) and the acoustic-vestibular ganglia area (AVG, Tuj1-positive), which would develop into sensory epithelium and the spiral ganglion, respectively. The OV and AVG areas were measured with the ImageJ software, and the data are presented as means ± SEM relative to control values (I, J). High magnification pictures were taken from the center of the otocysts (A4–H4), and the EdU-positive cells in each 63×high power field (HPF) were counted under the different conditions (K). * = *P*<0.05 compared to control, # = *P*<0.05 compared to RRD-251 10 µM, $ = *P*<0.05 compared to RRD-251 20 µM, and & = *P*<0.05 compared to U0126 30 µM (I–K). The changes in the ratios between the OV area and AVG area after treatment with U0126 and RRD-251 are shown in (L). * = *P*<0.05 and # = *P*<0.01 compared with controls (L). At least six otic vesicles per condition from three different experiments were evaluated, and statistical significance was estimated using one-way ANOVA. Scale bar = 50 µm.

When cultured otocysts were exposed to 10 µM, 20 µM or 40 µM RRD-251, the areas of the AVG and the OV were significantly decreased (Fig. 2A–D) with a dose-dependent reduction in the OV area to 80%, 68% and 36% (Fig. 2I) and a reduction in the AVG area to 59%, 35% and 25% (Fig. 2J) that of the DMSO-treated control otocysts. Furthermore, we assessed EdU incorporation in cultured otocysts to measure the rate of proliferation. The reduced proliferation caused by RRD-251 was confirmed by counting the number of EdU-positive cells per 63×High Power Field (HPF) at the center of the cultured otocysts (Fig. 2K).

The MAP kinase cascade is a well-established pathway that is involved in mitogen-induced cell proliferation and regulates pRb phosphorylation [18]. U0126 is a commonly used inhibitor of the MAP kinase pathway and specifically blocks ERK phosphorylation [19]. When cultured otocysts were treated with U0126, both the OV areas and AVG areas were significantly reduced in a dose-dependent manner (Fig. 2E–G). Increasing the concentration of U0126 from 30 µM to 50 µM led to a reduction in the OV area to 75% and 43% (Fig. 2I), respectively, and to a reduction in the AVG area to 67% and 56% (Fig. 2J), respectively, compared to the areas in the DMSO-treated control otocysts. Furthermore, the number of EdU-positive cells decreased along with the reduced OV and AVG areas at the center of the cultured otocysts (Fig. 2K).

Culturing the otocysts simultaneously with both U0126 (30 µM) and RRD-251 (20 µM) reduced the OV and AVG areas and the number of EdU-positive cells compared to 30 µM U0126 alone (Fig. 2H–K). This suggests a synergistic effect of the two inhibitors.

### The Ratios of the AVG and OV Areas Distinguish between the Two Pathways that Alter pRb Phosphorylation

**Figure 3:**
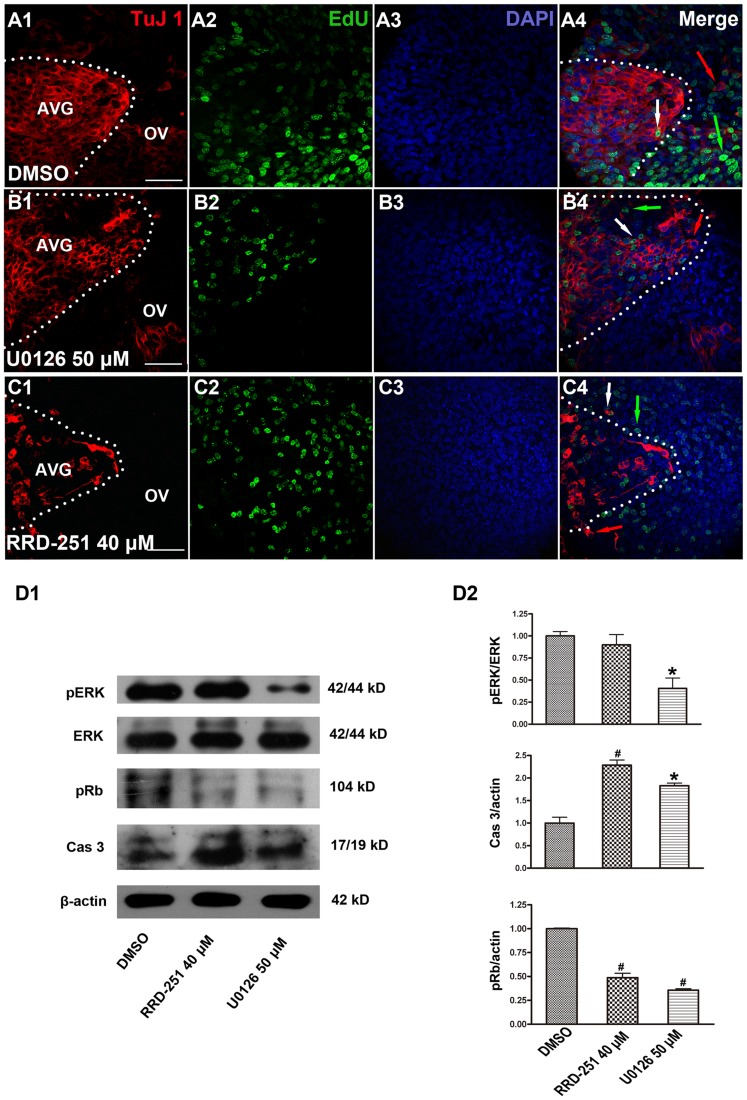
Sensory epithelium treated with U0126 or RRD-251. (A–C) Neuroblasts and progenitor cells of the sensory epithelium had distinct responses to the inhibition of the two independent pathways of pRb phosphorylation. (D1–D2) The explanted otic vesicles were cultured with DMSO, RRD-251 40 µM, or U0126 50 µM for 24 hours and then lysed to detect the level of phosphorylated and unphosphorylated ERK, pRb, and activated Caspase-3. β-actin was used as the loading control. Green arrows indicate the EdU-positive cells, red arrows indicate the Tuj1-positive cells, and the white arrows indicate the Tuj1 and EdU double-positive cells. At least three different experiments were evaluated, and the statistical significance was estimated using one-way ANOVA. * = *P*<0.05 and # = *P*<0.01 compared to controls. Scale bar = 50 µm.

Despite the fact that the AVG and OV most likely share a common origin, these cells experience distinct reactions to the abolishment of pRb phosphorylation through the two different pathways. Treatment with U0126 reduced the OV area to a greater extent than the AVG area, and the ratio of the OV and AVG areas was 2 compared to 3.5 in control otocysts treated with DMSO. Treatment with RRD-251, however, resulted in significantly reduced AVG area and correspondingly undifferentiated round-shaped otocysts, and the ratio of the OV and AVG areas increased dramatically to 4 (Fig. 2L). At a relatively high magnification, we found that there were EdU-positive cells in both the AVG and OV regions in control otocysts treated with DMSO (Fig. 3A). We observed fewer EdU-positive cells in the OV region in the U0126-treated group (Fig. 3B) and fewer EdU-positive cells in the AVG region in the RRD-251–treated group (Fig. 3C).

The effects on pRb phosphorylation by the two inhibitors were confirmed by western blot. U0126 significantly reduced ERK phosphorylation and pRb phosphorylation compared to controls, and the addition of RRD-251 to the otocyst culture significantly reduced the phosphorylation of pRb without affecting the phosphorylation of ERK (Fig. 3D1). The apoptosis of cultured otocysts was measured by the level of cleaved Caspase-3. Our data showed that when the cultured otocysts were exposed to U0126 or RRD-251 the levels of cleaved Caspase-3 were significantly increased (Fig. 3D1, D2).

### Disrupting the pRb-Raf-1 Interaction Leads to Increased Apoptosis during Early Otocyst Development

The early development of the inner ear requires a dynamic balance between the mechanisms regulating cell division, differentiation, and death [1]. Based on the signals from EdU, cleaved Caspase-3, and DAPI (to show the nuclei), we divided the cells in the cultured otocysts into three groups as a reflection of the balance among proliferation (EdU-positive and DAPI-positive), apoptosis (cleaved Caspase-3-positive and DAPI-positive), and quiescence (EdU-negative, cleaved Caspase-3-negative, and DAPI-positive). When the cultured otocysts were exposed to U0126, the number of cleaved Caspase-3–positive cells increased compared to the otocysts treated with DMSO alone (data not shown). This result confirms the data presented by Magarinos et al [2].

**Figure 4:**
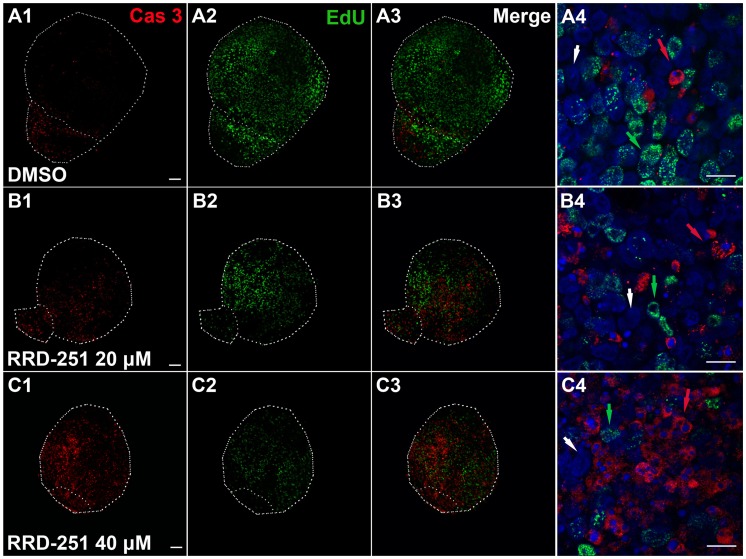
RRD-251 promoted apoptosis during the early development of otic vesicles. After treatment with DMSO (A1–A4), RRD-251 20 µM (B1–B4), or RRD-251 40 µM (C1–C4), the balance between proliferation, apoptosis, and quiescence during otic development was shifted. In the DMSO control group, nearly half of the cells incorporated EdU (48.78%), only 7.32% of the cells were Caspase-3–positive, and 43.9% of the cells in the otic vesicles were double negative (A4). After treatment with RRD-251, the portion of EdU-positive cells was reduced to 29.85% and 20.84% for doses of 20 µM (B4) and 40 µM (C4), respectively, and the portion of cleaved Caspase-3–positive cells was increased to 7.46% and 47.22%, respectively. Furthermore, compared with controls, the portion of double-negative cells was increased at the lower dose of RRD-251 (B4) to 62.69% and was reduced with the higher dose (C4) to 31.94%. Green arrows indicate the EdU-positive cells, red arrows indicate the cleaved Caspase-3–positive cells, and the white arrows indicate the double-negative cells. The differences between the three separate cell populations within each group were estimated using the χ^2^ test (*P*<0.001). Scale bar = 10 µm.

RRD-251 was developed as an antineoplastic drug that abolishes the phosphorylation of pRb by specifically disrupting the physical interaction between Raf-1 and pRb, and this has been shown to increase the apoptosis of tumor cells both in vitro and in vivo [20]. In our experiment, the addition of increasing concentrations of RRD-251 (20 µM and 40 µM) significantly increased the number of cleaved Caspase-3–positive cells. This was evident by areas of apoptotic cell death in which condensed nuclei were surrounded by cytoplasm containing active Caspase-3. Thus, the balance between proliferation, apoptosis, and quiescence during the early development of otocysts was shifted upon treatment with RRD-251, and the number of Caspase-3–positive cells was increased while the number of EdU-positive cells was reduced. The number of EdU and cleaved Caspase-3 double negative cells was increased with a lower dose of RRD-251, but this number was reduced when treated with a higher dose of RRD-251 (Fig. 4).

### Interfering with the Phosphorylation of pRb Affects Genes Involved in Cell Proliferation

**Figure 5:**
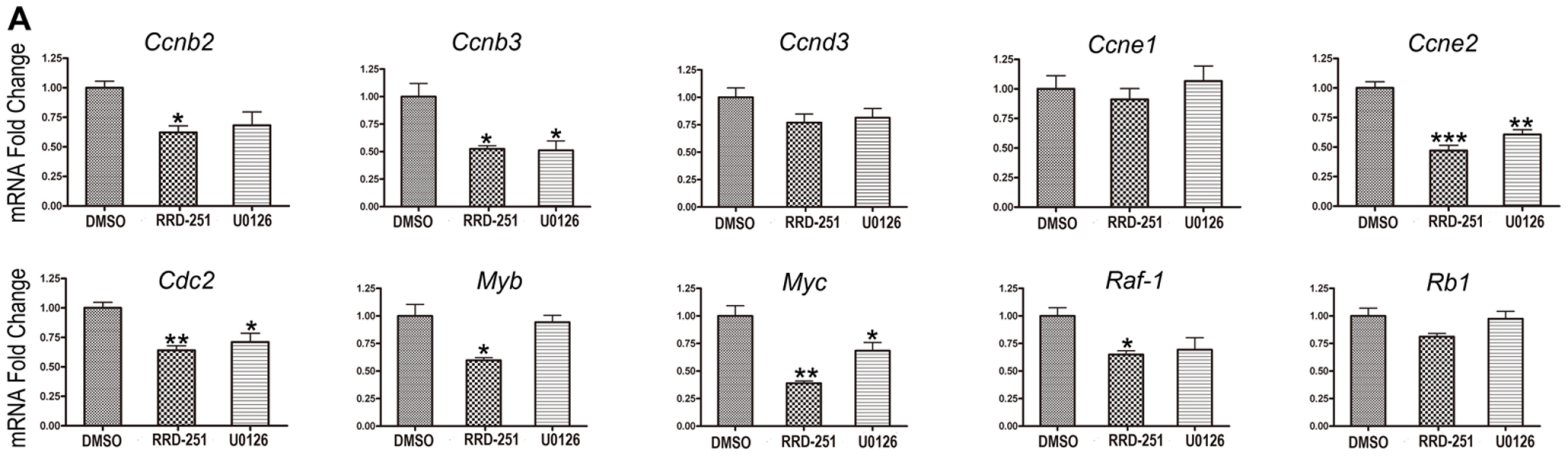
Expression of genes involved in cell cycle progression after the attenuation of pRb phosphorylation by inhibiting the two different pathways. The estimated gene expression was calculated as 2^−ΔΔCt^ and statistical significance was estimated using one-way ANOVA. * = *P*<0.05, ** = *P*<0.01, and *** = *P*<0.001 compared to controls.

In order to understand the mechanism behind the attenuated proliferation that results from abolishing the phosphorylation of pRb, we investigated the activity of cell cycle checkpoint genes. We found that the proliferation genes were downregulated to different extents. Among all of the genes tested, the expression of *Ccnb2*, *Ccnb3*, *Ccne2*, *Cdc2*, *Myb*, and *Myc* were significantly reduced when the otocysts were treated with RRD251, and the expression of *Ccnb3*, *Ccne2*, *Cdc2*, and *Myc* were significantly reduced when the otocysts were treated with U0126. Furthermore, we found that the expression of *Raf-1* was reduced in otocysts treated with RRD-251 but no change was seen in *Rb1* mRNA expression. We observed no difference in the expression of these two genes in otocysts treated with U0126 (Fig. 5).

## Discussion

Many lines of investigation have demonstrated pivotal roles for pRb in maintaining the postmitotic states of hair cells and supporting cells in the inner ear as well as promoting the survival of differentiated hair cells [5]–[7], [21]. We found that *Rb1* mRNA and pRb protein were expressed during the relatively early stages of inner ear development, and this suggests that the function of pRb goes beyond cell proliferation control and that the protein plays important roles during developmental processes in the inner ear. Furthermore, there was a significant amount of phosphorylated pRb, the inactive form of pRb, in the inner ear and this is consistent with the robust cell proliferation during early inner ear development. The highly expression of G1/S specific genes at HH18, which stage we were dealing with, suggested that there were more cells undergoing S Phase entry and DNA replication and provided a period to investigate the effects on proliferation by interfering the pRb phosphorylation.

Phosphorylation of pRb progressively attenuates pRb activity as a function of cell cycle progression, which is precisely regulated through a series of phosphorylation events [22], [23]. When the balance between the inactive pRb and active pRb forms during early inner ear development was shifted by exposure to U0126 and RRD-251, the progenitor cells with proliferative potential tended to withdraw from the cell cycle, stop growing, and undergo apoptosis. We demonstrated that the phosphorylation of pRb during the early stage of inner ear development could be regulated through two independent pathways, a MAP kinase cascade-mediated pRb phosphorylation and a Raf-1–induced pRb phosphorylation. According to the dose-dependent effect and the cumulative effect of a combination of U0126 and RRD-251, we concluded that both the mitosis signaling cascade and Raf-1 directly inducing pRb phosphorylation play vital roles in cell proliferation and cell survival in the inner ear. Furthermore, the fact that RRD-251 directly attenuated the phosphorylation of pRb and had no effect on the phosphorylation of ERK suggested that the binding of Raf-1 to pRb is independent of the MAP cascade pathway for the inactivation of pRb. The difference between the exposure to 20 µM and 40 µM RRD-251 was most likely due to a matter of the sequence of events leading to quiescence and then death.

RRD-251 has been shown to be effective at inhibiting the proliferation of cells harboring a wide variety of mutations in signaling cascades that inactivate pRb, but it does not affect cells carrying mutated *Rb1* or cells in which *Rb1* has been deleted. In addition, the specificity of this agent is demonstrated by the observation that the growth arrest caused by RRD-251 can be rescued by knockdown of the *Rb1* gene with a short hairpin RNA or by the overexpression of E2F1 [12].

pRb is required for cell-cycle exit of embryonic mammalian hair cells but is not required for hair cell fate determination and early differentiation. This specificity of the role of pRb provides a strategy of hair cell regeneration by manipulating pRb pathway. Because *Rb1* knockout cells are inviable, reversible manipulation of pRb activity might provide a new strategy for the regeneration of hair cells in the inner ear. It has been reported that Sonic hedgehog initiates cochlear hair cell regeneration through downregulation of pRb in postnatal rats [8]. Furthermore, supporting cells can be driven to re-enter the cell cycle by over-expressing cyclin D1 (a key component of the MAP cascade) in adult utricles [24]. Our results suggest a novel approach to regulating the function of pRb by enhancing the interaction of Raf-1 and pRb in the context of proliferation and hair cell differentiation. In addition, we found that *Rb1* mRNA was expressed in chicken otocysts at a relatively high level at the early HH18 stage, but pRb protein was detected at a low level at this stage. This discrepancy might be due to a delay in translation or to post-translational modification and regulation. The latter deserves to be investigated further as another approach for modifying the function of the *Rb1* gene.

In spite of a significant amount of data showing that the progenitors of sensory epithelium and spiral ganglion neurons originate from the same population of progenitor cells in the inner ear [25], [26], these cells still reacted differently to the abolishment of pRb phosphorylation through the two distinct pathways. Future lineage tracing experiments using transgenic animals or cell type specific interference by molecular techniques could address this question unequivocally.

It has been reported that pRb controls the G1 to S transition by repressing the transcriptional activity of the E2F protein family that is required for the expression of genes necessary for DNA synthesis and cell cycle progression [27], [28]. By inhibiting the phosphorylation of pRb, we found that both pathways that regulate pRb phosphorylation ultimately converge on cell-cycle gene regulation. In particular, the transcription of *Ccne2, Cdc2* and *c-Myc* was significantly reduced upon attenuation of pRb phosphorylation by inhibiting the two independent pathways. This result provides a greater understanding of the genetic networks and the key factors that regulate proliferation in the inner ear.

We conclude that phosphorylation of pRb is crucial for inner ear progenitor cell proliferation and survival during avian inner ear development. The interaction of Rb–Raf-1 plays an important role in the functional modification of pRb during the development of the inner ear, and this strengthens and extends the importance of the Rb–Raf-1 interaction that was previously shown to be important during hair cell regeneration in the neuromasts of zebrafish [29]. Our findings suggest a new strategy for modifying pRb by enhancing the interaction of Rb–Raf-1 in the context of proliferation and hair cell differentiation.

## References

[1] Kelley MW (2006) Regulation of cell fate in the sensory epithelia of the inner ear. Nat Rev Neurosci 7: 837–849. 17053809 10.1038/nrn1987

[2] Magariños M, Aburto MR, Sanchez-Calderon H, Muñoz-Agudo C, Rapp UR, et al (2010) RAF kinase activity regulates neuroepithelial cell proliferation and neuronal progenitor cell differentiation during early inner ear development. PLoS ONE 5: e14435. 21203386 10.1371/journal.pone.0014435 PMC3010996

[3] Swanson GJ, Howard M, Lewis J (1990) Epithelial autonomy in the development of the inner ear of a bird embryo. Dev Biol 137: 243–257. 2303163 10.1016/0012-1606(90)90251-d

[4] Lang H, Bever MM, Fekete DM (2000) Cell proliferation and cell death in the developing chick inner ear: spatial and temporal patterns. J Comp Neurol 417: 205–220. 10660898 10.1002/(sici)1096-9861(20000207)417:2<205::aid-cne6>3.0.co;2-y

[5] Sage C, Huang M, Karimi K, Gutierrez G, Vollrath MA, et al (2005) Proliferation of functional hair cells in vivo in the absence of the retinoblastoma protein. Science 307: 1114–1118. 15653467 10.1126/science.1106642

[6] Sage C, Huang M, Vollrath MA, Brown MC, Hinds PW, et al (2006) Essential role of retinoblastoma protein in mammalian hair cell development and hearing. Proc Natl Acad Sci USA 103: 7345–7350. 16648263 10.1073/pnas.0510631103 PMC1450112

[7] Yu Y, Weber T, Yamashita T, Liu Z, Valentine MB, et al (2010) In vivo proliferation of postmitotic cochlear supporting cells by acute ablation of the retinoblastoma protein in neonatal mice. J Neurosci 30: 5927–5936. 20427652 10.1523/JNEUROSCI.5989-09.2010 PMC2902201

[8] Lu N, Chen Y, Wang Z, Chen G, Lin Q, et al (2013) Sonic hedgehog initiates cochlear hair cell regeneration through downregulation of retinoblastoma protein. Biochem Biophys Res Commun 430: 700–705. 23211596 10.1016/j.bbrc.2012.11.088 PMC3579567

[9] Knudsen ES, Knudsen KE (2006) Retinoblastoma tumor suppressor: where cancer meets the cell cycle. Exp Biol Med (Maywood) 231: 1271–1281. 16816134 10.1177/153537020623100713

[10] Wang S, Ghosh RN, Chellappan SP (1998) Raf-1 physically interacts with Rb and regulates its function: a link between mitogenic signaling and cell cycle regulation. Mol Cell Biol 18: 7487–7498. 9819434 10.1128/mcb.18.12.7487 PMC109329

[11] Di Fiore R, D’Anneo A, Tesoriere G, Vento R (2013) RB1 in cancer: different mechanisms of RB1 inactivation and alterations of pRb pathway in tumorigenesis. J Cell Physiol 228: 1676–1687. 23359405 10.1002/jcp.24329

[12] Kinkade R, Dasgupta P, Carie A, Pernazza D, Carless M, et al (2008) A small molecule disruptor of Rb/Raf-1 interaction inhibits cell proliferation, angiogenesis, and growth of human tumor xenografts in nude mice. Cancer Res 68: 3810–3818. 18483265 10.1158/0008-5472.CAN-07-6672 PMC3233839

[13] Davis RK, Chellappan S (2008) Disrupting the Rb-Raf-1 interaction: a potential therapeutic target for cancer. Drug News Perspect 21: 331–335. 18836591 10.1358/dnp.2008.21.6.1246832 PMC2800199

[14] Singh S, Davis R, Alamanda V, Pireddu R, Pernazza D, et al (2010) Rb-Raf-1 interaction disruptor RRD-251 induces apoptosis in metastatic melanoma cells and synergizes with dacarbazine. Mol Cancer Ther 9: 3330–3341. 21139044 10.1158/1535-7163.MCT-10-0442 PMC3058238

[15] Hamburger V, Hamilton HL (1992) A series of normal stages in the development of the chick embryo. 1951. 42 pp. 10.1002/aja.1001950404 1304821

[16] Li H, Corrales CE, Wang Z, Zhao Y, Wang Y, et al (2005) BMP4 signaling is involved in the generation of inner ear sensory epithelia. BMC Dev Biol 5: 16. 16107213 10.1186/1471-213X-5-16 PMC1198226

[17] Li H, Liu H, Corrales CE, Mutai H, Heller S (2004) Correlation of Pax-2 expression with cell proliferation in the developing chicken inner ear. J Neurobiol 60: 61–70. 15188273 10.1002/neu.20013

[18] Shukla S, Gupta S (2007) Apigenin-induced cell cycle arrest is mediated by modulation of MAPK, PI3K-Akt, and loss of cyclin D1 associated retinoblastoma dephosphorylation in human prostate cancer cells. Cell Cycle 6: 1102–1114. 17457054 10.4161/cc.6.9.4146

[19] Favata MF, Horiuchi KY, Manos EJ, Daulerio AJ, Stradley DA, et al (1998) Identification of a novel inhibitor of mitogen-activated protein kinase kinase. J Biol Chem 273: 18623–18632. 9660836 10.1074/jbc.273.29.18623

[20] Dasgupta P, Sun J, Wang S, Fusaro G, Betts V, et al (2004) Disruption of the Rb–Raf-1 interaction inhibits tumor growth and angiogenesis. Mol Cell Biol 24: 9527–9541. 15485920 10.1128/MCB.24.21.9527-9541.2004 PMC522224

[21] Weber T, Corbett MK, Chow LML, Valentine MB, Baker SJ, et al (2008) Rapid cell-cycle reentry and cell death after acute inactivation of the retinoblastoma gene product in postnatal cochlear hair cells. Proc Natl Acad Sci USA 105: 781–785. 18178626 10.1073/pnas.0708061105 PMC2206613

[22] Weinberg RA (1995) The retinoblastoma protein and cell cycle control. Cell 81: 323–330. 7736585 10.1016/0092-8674(95)90385-2

[23] Buchkovich K, Duffy LA, Harlow E (1989) The retinoblastoma protein is phosphorylated during specific phases of the cell cycle. Cell 58: 1097–1105. 2673543 10.1016/0092-8674(89)90508-4

[24] Loponen H, Ylikoski J, Albrecht JH, Pirvola U (2011) Restrictions in cell cycle progression of adult vestibular supporting cells in response to ectopic cyclin D1 expression. PLoS ONE 6: e27360. 22073316 10.1371/journal.pone.0027360 PMC3206952

[25] Li H, Liu H, Heller S (2003) Pluripotent stem cells from the adult mouse inner ear. Nat Med 9: 1293–1299. 12949502 10.1038/nm925

[26] Martinez-Monedero R, Yi E, Oshima K, Glowatzki E, Edge ASB (2008) Differentiation of inner ear stem cells to functional sensory neurons. Devel Neurobio 68: 669–684. 10.1002/dneu.20616 18278797

[27] Cobrinik D (2005) Pocket proteins and cell cycle control. Oncogene 24: 2796–2809. 15838516 10.1038/sj.onc.1208619

[28] Stengel KR, Thangavel C, Solomon DA, Angus SP, Zheng Y, et al (2009) Retinoblastoma/p107/p130 pocket proteins: protein dynamics and interactions with target gene promoters. 284: 19265–19271. 10.1074/jbc.M808740200 PMC2740551 19279001

[29] Lin Q, Li W, Chen Y, Sun S, Li H (2013) Disrupting Rb-Raf-1 interaction inhibits hair cell regeneration in zebrafish lateral line neuromasts. Neuroreport 24: 190–195. 23381351 10.1097/WNR.0b013e32835e3279

